# Exploring the use and experience of an infant feeding genogram to facilitate an assets-based approach to support infant feeding

**DOI:** 10.1186/s12884-020-03245-8

**Published:** 2020-09-29

**Authors:** Gill Thomson, Jenny Ingram, Joanne L. Clarke, Debbie Johnson, Heather Trickey, Stephan U. Dombrowski, Pat Hoddinott, Kirsty Darwent, Kate Jolly, on behalf of the ABA Research Group

**Affiliations:** 1 Maternal and Infant Nutrition and Nurture Unit (MAINN), University of Central Lancashire, Preston, UK; 2 School of Education, Health and Social Studies, Dalarna University, Högskolegatan 2, Falun, Sweden; 3 Centre for Academic Child Health, University of Bristol, Bristol, UK; 4 Institute of Applied Health Research, Murray Learning Centre, University of Birmingham, Birmingham, B15 2TT UK; 5 DECIPHER, Department of Social Medicine, Cardiff University, Cardiff, UK; 6 Faculty of Kinesiology, University of New Brunswick, Fredericton, Canada; 7 Division of Psychology, University of Stirling, Stirling, UK; 8 Nursing, Midwifery and Allied Health Professions Research Unit, University of Stirling, Stirling, UK; 9 Faculty of Science and Sport, University of Stirling, Stirling, UK

**Keywords:** Breast feeding, Bottle feeding, Social support, Women, Assets based, Genogram, Infant feeding

## Abstract

**Background:**

A lack of perceived social support influences women’s infant feeding behaviours. The Infant Feeding Genogram is a visual co-constructed diagram which details people/services that can provide support to women and can facilitate a connection between mothers and their existing assets landscape. The aim of this study is to explore women’s and infant feeding helpers’ experiences and use of an infant feeding genogram delivered to the intervention group of the “Assets-based infant feeding help Before and After birth (ABA)” randomised feasibility trial.

**Methods:**

103 primiparous mothers aged 16+ years were recruited to the trial (trial registration number) in two sites (Site A and Site B) with low breastfeeding prevalence in the UK. Infant feeding helpers (IFHs) co-constructed a genogram at the first antenatal meeting for the intervention group (*n* = 50), and then provided proactive, woman-centered support from ~ 32 weeks gestation to up to 5 months postnatal. Infant feeding helpers' and women’s experiences of the infant feeding genogram were collected via interviews or focus groups. Completed genograms were shared with researchers. Content analysis of the genograms and qualitative data from the interviews and focus groups were analysed thematically.

**Results:**

Data comprised 32 completed genograms, and qualitative insights from all 13 infant feeding helpers (two focus groups; 4 interviews) and interviews with a purposive sample of 21 of 50 intervention group women between 4 and 21 weeks after birth. Content analysis of the genograms highlighted variations, with more personal, individualised genograms completed at Site B compared to Site A. The perceived impact of the genogram was related to the IFHs’ application of the tool. The genogram was either used as intended to raise women’s awareness of available assets and motivate help-seeking behaviour, or as a data collection tool with limited perceived utility. Negative and positive unintended consequences of genogram use were highlighted.

**Conclusions:**

The genogram has the potential to offer a woman, family and community-centred approach that focusses on building assets for infant feeding. However, variations in genogram application indicate that revised training is required to clarify the purpose and ensure it is used as intended.

**Trial registration:**

ISRCTN ISRCTN14760978; Registered 30 January 2017.

## Background

Infant feeding is a key public health issue. While there is a wealth of evidence that breast/breast-milk feeding optimizes infant and maternal health [1], the UK has one of the lowest breastfeeding rates globally [2]. Breastfeeding rates are also socially patterned, being substantially lower within socially deprived communities [2]. Most UK mothers introduce formula milk at some stage in their feeding journey, and within an overall framework of a public health policy to promote breastfeeding there is also a public health focus on safe and responsive formula feeding. Mothers commonly make errors in reconstitution of formula milks, with a tendency to over-concentrate feeds [3] and while most understand the guidelines for making up formula feeds, this knowledge has not always translated into compliance [4].

Social and cultural factors are a powerful influence on women’s infant feeding decisions [5, 6], with evidence that social and family support is more important than support provided by healthcare providers [7]. Family support can help to increase breastfeeding confidence and practical breastfeeding skills. For instance, a longitudinal study of 203 mothers found that mothers who continued breastfeeding rated their partner and mother as having more pro-breastfeeding views [8]. However, from a counter perspective, unsupportive behaviours and negative attitudes from families and personal networks can undermine women’s self-efficacy and can lead to non-breastfeeding or early breastfeeding cessation [9–11]. The need for family-centred approaches and supportive personal and community networks (i.e. breastfeeding groups, support from like-minded peers) to provide emotional and practical support are reported [6, 12–14].

Over the last decade, assets-based approaches to public health have emerged, which aim to address some of the social and cultural barriers to positive health. An assets-based approach aims to empower people and communities to think about and use the assets they have at their disposal [15, 16] such as the skills, knowledge and passion of supportive individuals or local services [15–17]. Such approaches are designed to operate on an intrinsic and extrinsic basis, such as via developing self-esteem and coping skills and creating stronger connections and relationships [15, 16, 18]. Although currently there is little practical guidance as to how assets-based approaches can be delivered by frontline staff. One tool which could facilitate an assets-based approach to support infant feeding is the Infant Feeding Genogram. The use of genograms originates within systemic family therapy [19]. Darwent and colleagues [20] developed an Infant Feeding Genogram that involves a trained facilitator working with a mother to provide a visual representation of the woman’s family infant feeding history, the people who can provide support, and the interconnections between them. In Darwent’s study, she used the genogram to explore the experiences of women who were the first to breastfeed in their family. Women found the genogram to be acceptable and it helped them identify sources of breastfeeding support; although the need for further research was highlighted [20].

In the “Assets-based infant feeding help Before and After birth (ABA)” feasibility trial [21–23] a modified version of Darwent’s infant feeding genogram [20] was used to increase women’s assets for infant feeding. This paper explores infant feeding helpers and women’s use and experience of the genogram as an intervention component in the ABA feasibility trial.

## Methods

### Intervention design

While full details of intervention delivery and recruitment into the feasibility trial are reported elsewhere [21, 22] – a summary is provided as follows. The ABA intervention was an Infant Feeding Helper (IFH) peer support service delivered from ~ 32 weeks gestation to ~ 5 months postnatal. ABA was designed to be assets-based by including genogram completion and providing women with an assets leaflet that mapped local/national sources of infant feeding support. It was based on behaviour change theory and included two core behaviour change techniques (BCTs) [24, 25] - ‘restructuring the social environment’ and ‘social support (unspecified)’. Both BCTs underpinned the use of the genogram in terms of this tool’s perceived utility to increase awareness of the skills, networks and connections available to support infant feeding. The ABA support was also intended to be woman-centred in that the beliefs, goals and values of the woman being supported were paramount; women were supported to achieve their feeding goals, however they intended to feed their babies [26].

The genogram was used at the first contact between the IFH and woman (and her partner/family member if the woman desired) at ~ 32 weeks gestation. The contact was scheduled for a one-hour face to face meeting to discuss infant feeding, complete the genogram, and to discuss/provide the assets leaflet. The IFHs then continued proactive support (primarily via telephone/text) up to ~ 5 months postnatal [21].

### Study site/IFH recruitment

The ABA study was undertaken at two geographical sites in England. Site selection was based on low breastfeeding (initiation and continuation) rates and for operating peer support services in place. Existing peer supporters were recruited to become ABA IFHs. Site A was an urban setting with IFHs (*n* = 6) recruited from a paid breastfeeding peer support service. Site B was a suburban setting, with IFHs (*n* = 7) recruited from a volunteer-based peer support service. All the IFHs had accessed accredited peer supporter training from their host organisation.

### IFH training – genogram completion

IFHs received six hours training into the assets-based, woman-centred intervention. It was initially delivered to Site A IFHs, allowing for adjustments to timings of the programme to be made when delivered in Site B. Originally it was intended that Darwent’s four-stage process was to be used as the basis for genogram training [20]. This involved: ‘mapping family structure’ - detailing women’s partner, children, parents, grandparents; ‘mapping infant feeding information’ - adding colours to clearly depict who has/is currently breastfeeding; ‘recording strong family bonds or conflict’ - including symbols to denote relationship patterns; ‘adding other important people’ - such as friends and community sources who can support infant feeding. However, the study team felt asking IFHs to comply with all these stages could be perceived as overly complicated (from an IFH and woman perspective). Furthermore, it was anticipated that the methodology itself would be difficult to embed within the skill-set of IFHs given the limited training time, where only 30 min was available to teach the genogram concept. The study team therefore decided to train the IFHs (via didactic and role play methods) to apply the *principles* of the genogram without the full four-step methodology. IFHs were shown how to work with the women they supported to draw a visual map, beginning by placing the woman herself at the centre and then co-producing a surrounding network of meaningful relationships. Strength or significance of relationships could be identified via the thickness of lines linking people to the woman. In this way, a visual representation of core information could be produced without the need for colours or symbols to depict the nature or quality of the relationships. The IFHs were advised that the focus was to have an open conversation with women to explore the infant feeding experiences of those around her as well as to identify those who would be available to support her in line with her own infant feeding intentions, with the genogram summarising this information in a simple diagram. Instruction on how the IFHs could support women who faced generational or attitudinal differences in infant feeding support was also provided. This included encouraging women to think about who could provide positive support, and to direct women to use the assets leaflets provided as part of the ABA intervention. The intention was that a copy of the genogram would be retained by the woman and IFH.

A suggested script was provided to the IFHs to be used as a basis for introducing the genogram at the antenatal meeting:

*‘We know that having friends and family who can offer you support when you have a new baby can make it easier to feed the way you want. If it’s okay, I would like to have a chat about your family and friends to find out how they’ve fed their own babies and how they feel about infant feeding. In this way, we can discover who might offer you the best support with feeding when you’ve had your baby. It can be helpful to draw a “Genogram” to show all these people on a piece of paper. It is like a family tree and can help identify who your key supportive people might be.’*

There was no specific instruction provided to the IFHs about ongoing use of the genogram with the woman after it had been completed, but they were encouraged to take a picture on their phone and use it if useful in subsequent contacts.

### Recruitment

Women were eligible to participate in the ABA feasibility trial if they were aged 16+ years and were pregnant with their first child. Community midwives provided women with study information at ~ 25–28 weeks gestation and then a researcher approached women at antenatal clinics to gain informed consent. The intention was to recruit at least 100 women to the study (50 per site); with insights from some of the women in the intervention arm (*n* = 50) being reported in this paper.

### Data collection

Data contributing to the evaluation of the use of genograms comprised: a) completed genograms from 11 IFHs (*n* = 32), with information anonymised via use of pseudonyms; b) semi-structured face to face interviews (see Supplementary File 1 for interview schedule) with a purposive sample of 21 women who had been offered the ABA intervention. Participants were selected to capture a range of ages, feeding experiences and levels of engagement with the ABA intervention. All interviews took place at a single time point when the infants were aged between four and 21 weeks; c) focus groups and telephone interviews with all the 13 IFHs (see Supplementary File 2 for focus group/interview schedule). All interviews/focus groups contained questions that explored women’s/IFHs views and experiences of the genogram, were audio recorded and transcribed in full.

Data collection and analysis was undertaken by four experienced qualitative researchers (GT, JI, JC, DJ) from psychology, midwifery, public health and health services research backgrounds and two have a long history in the research/evaluation of breastfeeding peer support provision.

### Data analysis

Originally, we developed a coding framework and undertook a thematic approach [27] to identify women’s and IFHs experiences of the entire ABA intervention. For the purposes of this paper we re-analysed interview/focus group data relating to women’s and IFHs’ views and experiences of the genogram and analysed all completed genograms shared with the research team. This involved content analysis of the types and quality of data contained within the completed genograms, and further use of Braun & Clark’s thematic approach to analyse the interview/focus group data. This involved line by line coding, with codes mapped into themes on an iterative basis until all data were adequately represented [27]. GT led on data analysis, with all decisions discussed and shared within the wider team for consensual validation.

### Ethics

Ethical approval was received from South West – Cornwall and Plymouth Research Ethics Committee (16/SW/0336).

## Results

**Fig. 1:**
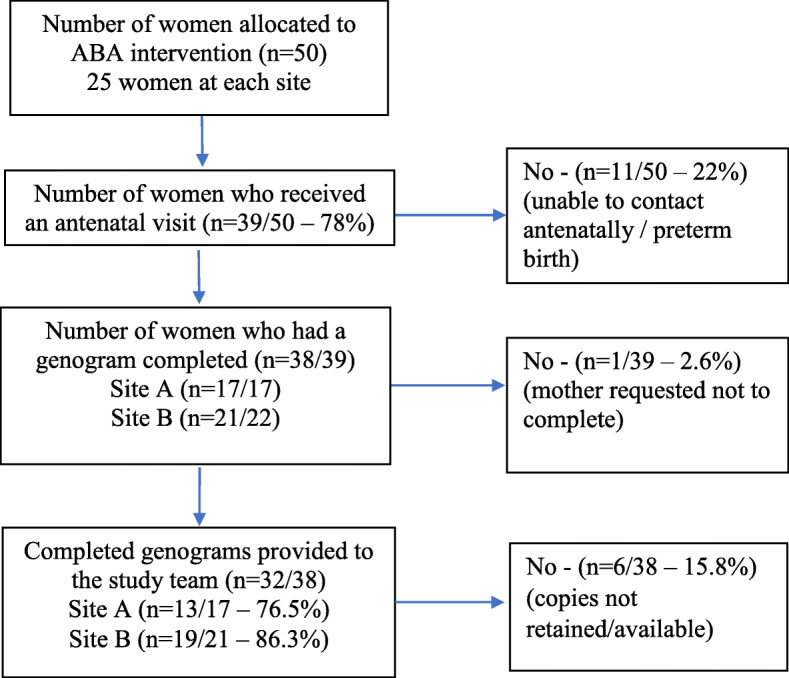
Flowchart of genogram completion and availability

Overall, 103 women were recruited to the ABA study – with insights from some of the women from the intervention arm (*n* = 50) reported in this paper. In Fig. 1 we provide an overview of the number of genograms that were completed and available for evaluation purposes. In summary, 39 of the 50 intervention women (78%) received an antenatal visit and 38 had a genogram completed (as detailed within the IFH records). Of the 38 completed genograms, 32 were submitted to the study team; 13 from Site A and 19 from Site B.

All 13 IFHs took part in either one of two focus groups (*n* = 9) or a telephone interview (*n* = 4), and 21 intervention women, all of whom completed a genogram, took part in a face-to-face interview. These women were aged between 19 and 37 years, and the majority were of a White British ethnicity and worked in a paid capacity. In Table 1 we provide characteristics of the women who a) took part in the intervention, b) were interviewed and c) had a genogram completed, with no marked variations identified.

**Table 1:** Characteristics of women who took part in the intervention, were interviewed and who had a genogram completed

Characteristic	All intervention women (***n*** = 50)	Intervention women interviewed (***n*** = 21)	Intervention women with genogram available (***n*** = 32)
Maternal age at baseline years (mean, SD)	28.6y (SD 5.2)	29.9y (SD 5.3)	28.7y (SD 5.3)
Ethnicity – White British, n (%)	43 (86.0%)	17 (81.0%)	28 (87.5%)
Employment - paid work, n (%)	40 (80.0%)	18 (85.7%)	26 (81.3%)
Baby age at interview (mean)	–	86.3 days	–
Any breastfeeding at 8 weeks	24/48 (50.0%)	12/21 (57.1%)	19/30 (63.3%) Missing = 2
Any breastfeeding at 6 months	18/39 (46.2%)	9/20 (45.0%) Missing = 1	16/29 (55.2%) Missing = 3

**Fig. 2:**
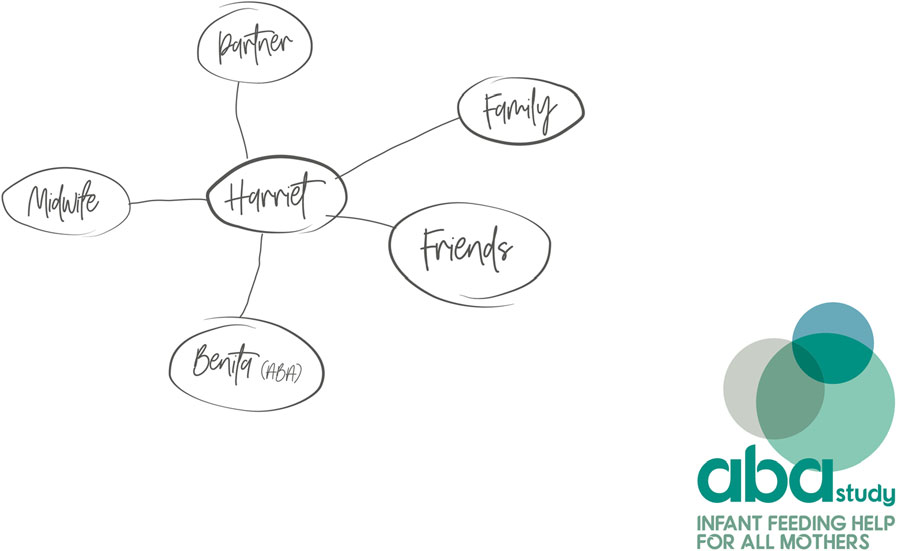
Type 1

**Fig. 3:**
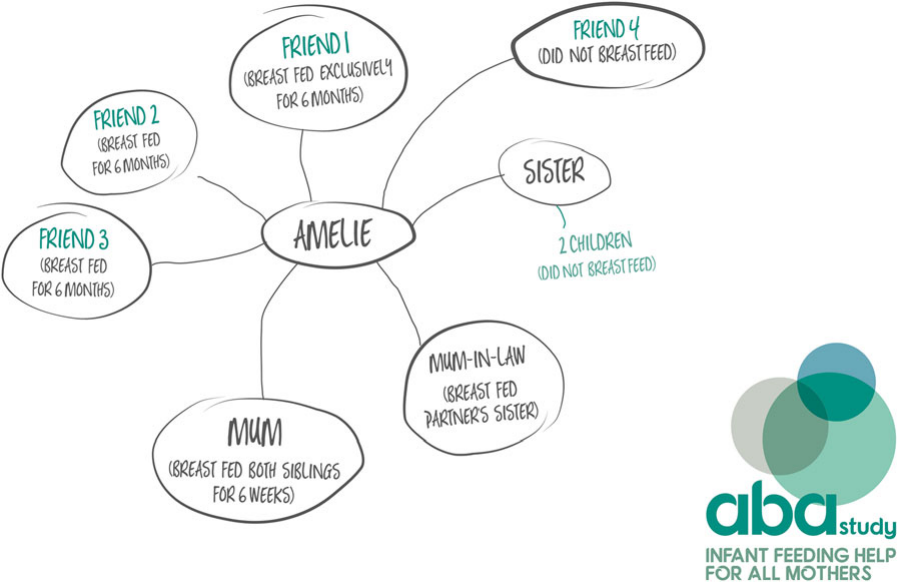
Type 2

**Fig. 4:**
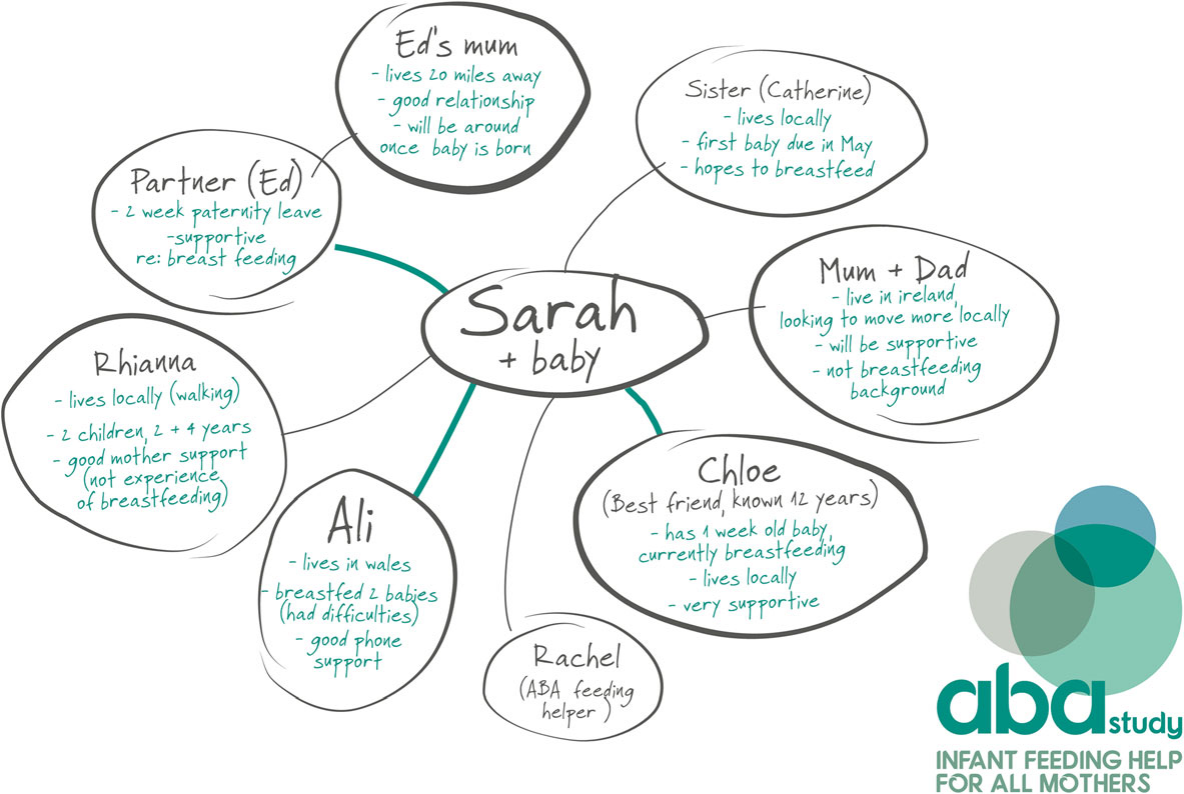
Type 3

**Fig. 5:**
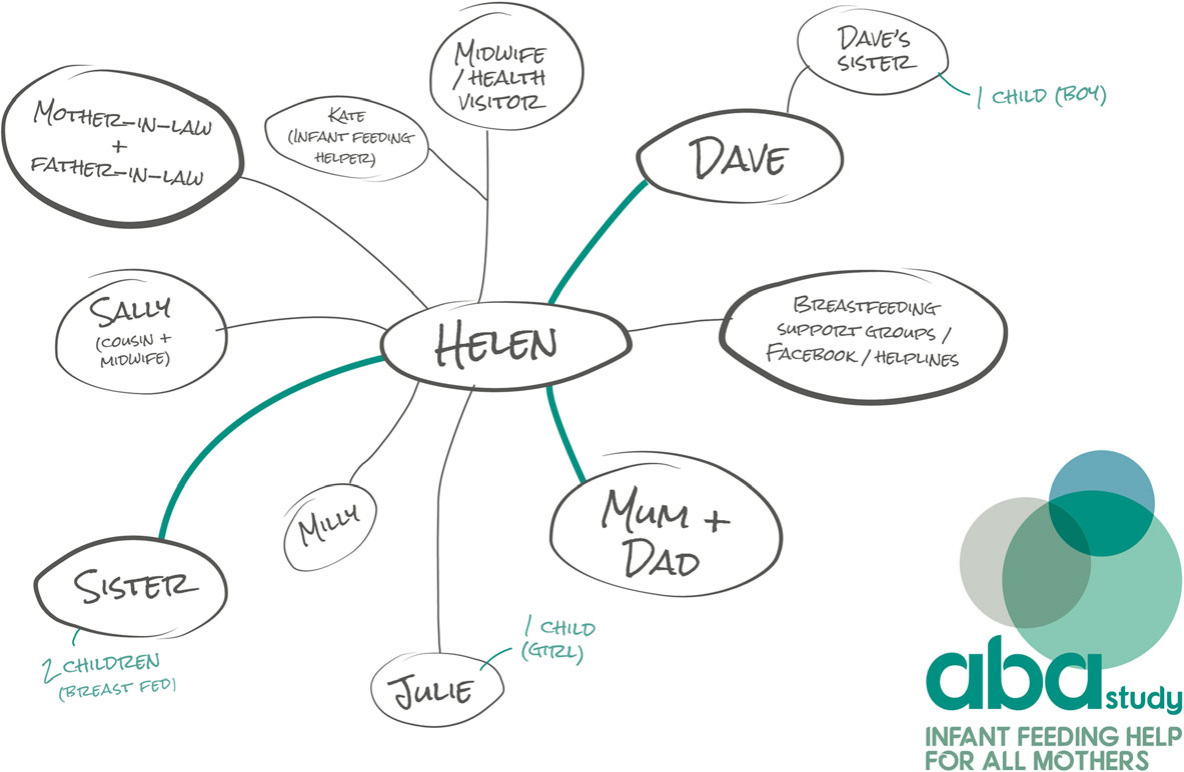
Type 4

While content analysis of the genograms highlighted wide variations, we defined four different genogram types. In Table 2 we provide a summary of the four different types of genogram completed by site and IFH; an example anonymised genogram for each type is also provided for illustrative purposes. Type 1 (Fig. 2) (*n* = 2/32) used categories of supporters (e.g. friend, family), provided no infant feeding details or quality of feeding support. Type 2 (see Fig. 3) (*n* = 11/32) generally detailed the supporters names (as opposed to categories), offered some information on infant feeding backgrounds/experiences, but no insights into the expected quality of support. Type 3 (Fig. 4) (*n* = 7/32) provided names of the supporters, rich insights into the supporters' infant feeding backgrounds and types of expected support, most contained information on the geographical location of the supporters and detailed the IFH as an additional form of support. Finally, Type 4 (Fig. 5) (*n* = 12/32) used the names of the women’s nominated supporters, provided some information on infant feeding and quality of expected support and detailed a wide range of community assets (e.g. groups, health professionals, IFHs). On a few occasions (notably Types 3 and 4), IFHs used colours (e.g. to depict different types of supporters, friends, family, etc) and thicker lines to depict the strength of the expected support from the different supporters. Overall, the analysis highlighted differences across the sites with Site A IFHs constructing Type 1 or Type 2 genograms and Site B creating Type 3 or Type 4.

**Table 2:** Typology of genogram completion (*n =* 32) by site and IFH

Genogram type	Frequency	Site	IFH
**Type 1 (see** Fig. 2**)** Supporter categories No feeding details No feeding support quality	2	A	IFH 1 (*n* = 1) IFH 6 (*n* = 1)
**Type 2 (see** Fig. 3**)** Some supporter names Some feeding information No feeding support quality	11	A	IFH 1 (*n* = 2) IFH 2 (*n* = 4) IFH 3 (*n* = 2) IFH 4 (*n* = 2) IFH 6 (*n* = 1)
**Type 3 (see** Fig. 4**)** Use named supporters Rich insights into infant feeding information Majority contain information on geographical location of support Expected quality of infant feeding support detailed	7	B	IFH11 (*n* = 3) IFH 10 (*n* = 4)
**Type 4 (see** Fig. 5**)** Named supporters Some infant feeding information Details of IFH and wider support networks Quality of infant feeding support indicated^a^	12	B	IFH 7 (*n* = 2) IFH 9 (*n* = 3) IFH 11 (*n* = 1) IFH 12 (*n* = 2) IFH 13 (*n* = 4)

^a^ Demonstrated by the thickness of lines to individual supporters

In the following sections we draw on the different genogram types across the sites, together with the IFHs and women’s qualitative data to present four themes: ‘building and enhancing networks of support’; ‘promoting positive wellbeing’; ‘perceived lack of value and utility’; and ‘unintended consequences’.

### Building and enhancing networks of support

A specific purpose of the genogram was to identify and raise awareness of extrinsic assets for infant feeding, such as the knowledge and skills of family and community members and wider community and wider resources. Type 3 and Type 4 genograms tended to contain more detailed insights (such as a wider range of community assets and infant feeding backgrounds of the named supporters). In turn, women from Site B referred to how the genogram had helped them to think about, e.g. ‘*my support pathways a bit more’*, and served as an aide-memoire of available support; *‘there’s a few people that she reminded me of actually’*, as well as extending the support they had available:

I’m not on my own, and that did help, because she illustrated that for me, and there was her, she was part of that support group, she was part of that support network as well. She was another person I didn’t have before. (P25, Site B).

One IFH also provided a key example of using the genogram as an assets-based tool in how she responded to a woman’s revelation of limited support to emphasise the wider networks of support that were available, should these be required:

She had no family or friends support with the breastfeeding, she was a bit reluctant. [ … ] she was getting a bit tense to say that well I’ve got no support and how am I going to manage to do this breastfeeding? But we talked around that and then we talked around the leaflet, the breastfeeding support, and I gave her our leaflet to say that once the baby is born just give us a ring and we can come and support you until about eight weeks after the baby is born and things like that, and I think that made her a bit more at ease. (IFH2, Site A, Interview)

Physical copies of the genograms were not used in future helper-mother contacts. However, some Site B mothers specifically referred to retaining and using a visual memory of the genogram to remind them of available support, i.e. ‘*in my head I’ve gone to it as a diagram since, I thought actually who else was on it, who else could I ask*’. Furthermore, a few of the Site B IFHs mentioned how they had used the names of the women’s supporters (from their phone pictures) to help direct them into available and appropriate assets:

I personally did when I was texting them or speaking to them because it helped me remember who they said their partners were, or if they had a certain relative that was significant in their life, so I would refer back to them and say is your sister [name] is she still popping round? (IFH 13 Site B, Interview)

Type 3 genograms tended to include information about the geographical location or proximity of available support. One woman specifically considered how this had helped her envisage the immediacy of available support, and enhanced her appreciation of who she could rely on:

It just made me rethink and evaluate how much I appreciate having some family closer by, because all of [partner]‘s family are local but all mine are spread out round the world. (P23, Site B)

Several mothers considered the genogram had not influenced them to seek out support, but this could be due to a lack of need, or challenges associated with new parenting, one mother reported *‘it was a good exercise to do at the time, but then everything has gone a bit to pot since’*. For others, the genogram was reported to have enhanced existing networks by encouraging women to seek help from known individuals who they would not necessarily have considered as a supporter, i.e. *‘I didn’t think of her [sister in law] as somebody to ask, and actually I’ve asked quite a few questions of her’*, as well as women seeking out support from multiple sources, e.g. friends, family, and neighbours:

I drew a feeding diagram with a network of people that could help, and I’ve got next door has got two young children, and they were really helpful, she’s lush, she’s really helpful, and I’ve got a couple of friends that have got young babies that I drop the odd text to saying is this normal? I’m in a WhatsApp group with some of the antenatal girls, we’re meeting up tomorrow for the first time actually, and we’ve been texting each other saying how is it going and talking about things, so that’s been good. (P20, Site B)

These women referred to how these conversations had been *‘useful’* and *‘interesting’* which for one related to eliciting divergent realities of breastfeeding amongst older and younger generations:

Yeah, so speaking to friends that have been through similar and I found it interesting that the majority of my friends of a similar age have found breastfeeding really very difficult in terms of either pain or other people have had milk supply issues, but the majority of people of my mum’s generation seem to have found it really very easy, no talk of pain. (P4, Site A)

### Promoting positive wellbeing

Women across both sites reported how completing the genogram had made them feel more *‘relaxed*’, ‘*confident’* and *‘more at ease’* about infant feeding. Genogram completion enhanced maternal wellbeing for some, such as through women feeling *‘lucky’* about the extent of support available to them:

It was good to think about it, made me realise how lucky I am to have fantastic family and friends and neighbours nearby (P4, Site A)

Women referred to how genogram completion had helped appease their concerns by raising awareness of valuable and available assets:

When she told me I thought oh we are going to finish really soon because I am all alone here with my husband, and it was not, because really you start thinking and you say oh no but I have this friend, I have that friend, I have this neighbour, so really it was a good experience. (P27, Site B)

Which for some, helped to reduce their perceived sense of social isolation:

It just made me realise, I was like oh okay, not as alone as I thought, because I think as a single mum I was like oh, but no, felt better (P24, Site B)

A few women referred to how the genogram had directly enhanced their confidence to seek out support. For instance, one woman alluded to how the genogram had helped her re-frame seeking support as a strength to achieve her infant feeding goals:

I think it was nice to see visually actually what I had around me to make it work, and one thing with a baby is actually it’s quite hard sometimes. I’ve always been very independent but it’s actually holding your hands up and going actually no I do … going to my parents actually, no I do need some help tonight. (P19, Site B)

The positive impact of the genogram on women was also echoed by some of the IFHs at both sites. These helpers considered the genogram to have provided women with reassurance as to the amount of support available to them:

I think they all felt reassured when they finished it. [ … ] I think because they probably hadn’t thought about how much support they had actually got, and it was a time to just focus on the support that they have got around them, and they all seemed quite happy afterwards. So that was really good. (IFH 10, Site B, Focus Group)

### Perceived lack of value and utility

As reflected in Table 2 above, Site A IFHs were less likely to record information on the supporters infant feeding experiences (e.g. Type 1 and Type 2). This may relate to women not knowing this background detail, or the genogram being utilised as a breastfeeding, rather than the intended ‘infant feeding’ tool. This was reflected in IFHs concerns of how discussions of formula milk would be reinforcing: i.e. *‘one that was formula feeding it* [genogram] *again affirmed why she was formula feeding’* and confirmed in women’s accounts; *‘I explained that I didn’t really have anyone close to me that had breastfed’*. The lack of information may also be associated with the IFHs views that infant feeding is a sensitive topic to be treated with caution as well as a low perceived value of the genogram. For instance, one IFH from Site A explicitly stated, *‘I didn’t like it* [genogram completion]’. She expressed her negativity towards asking women about other people’s feeding histories as it was perceived to be ‘*too personal’*, and repeated efforts to capture this detail was equated with *‘asking for too much information’*:

If somebody said to me what did your partner do [feeding] and to be honest I don’t really know, it doesn’t really bother me, and some people are like that as well, doesn’t matter which background they have come from, they may not have that knowledge, but you’re asking them too much information. [ … ] Because sometimes what happens is you know when you’re having a general conversation with the mum anyway she has probably brought all that up already [ … ] And then you throw in that genogram and you think well she’s already done that, so where do I include all that in now? And then what I had to do is okay I said, “This is a part of the actual study so like you said that your partner did breastfeed … ” I had to remember that and think like okay she’s already done that, rather than her to repeat it again. (IFH5, Site A, Focus Group)

This example highlights how the IFH assumed her negative views would be shared and clearly demarcated differences about talking to women about available support and constructing a diagram for the *‘actual study’* (in other words, the genogram being completed for research purposes only). Such sentiments, and lack of adherence to the underpinning ethos of the genogram was also reflected by other Site A IFHs who, e.g. considered the genogram to be a *‘pen and paper’* exercise; with one of the completed genograms detailed within a case-file record, rather than a stand-alone document to be left with the woman. The genogram not being completed as intended (i.e. as a tool for a meaningful discussion, raise awareness of assets) was also echoed in some of the women’s accounts. Here the woman equates genogram completion as a method to transmit information for the benefit of the IFH, and expresses a sense of disappointment as to how little information she could *‘give her* [IFH] *out of it’*:

I don’t know, I didn’t really … I already knew a lot of my friends were bottle feeding, I only knew one person who was breastfeeding at the time, I knew my mum had breastfed but everybody else I know had all bottle fed sort of thing, so it didn’t make much difference really. I just knew that my mum and one of my friends had breastfed but everybody else bottle fed, and that was all I could really give her out of it sort of thing. (P6, Site A)

The finding that categories rather than named supporters were used in Site A genograms may also indicate a lack of meaningful discussion, and reflect why some Site A women had little, or vague memories in undertaking this exercise:

They were here about an hour and I really don’t remember what we spoke about for an hour, because they just drew this diagram and then left. (P11, Site A).

Some women from both sites questioned the validity of the tool, as e.g. ‘*I already knew’* who was available to provide support. Tentative views on the influence of the genogram on women’s use of their local assets was highlighted by IFHs on both sites - with one offering a hesitant 50/50 success rate:

I don’t know, I think as I say it depends on the person that you see really, so if it was me I suppose taking part in it I wouldn’t necessarily feel that it would benefit me, because I know who I’ve got to support me, but maybe if you were in a different situation it might be beneficial to think about who else there is around, and you talk about the groups that are around a little bit and you remember you’ve got your midwife or your health visitor or whatever. So I’m not sure, I’m a bit 50/50 on it. (IFH 12, Site B, Interview)

While, as indicated above, most mothers considered that they did not need to revisit the genogram as it had provided a visual map of available support – the fact that some IFHs had not considered continued use of the genogram is potentially indicative of its perceived lack of value:

I never thought of that to be honest [ongoing use of the genogram in IFH-woman contacts], but I suppose I would think they would have come to you [IFH] after they had been to those support points, I would have thought. (IFH1, Site A, Interview)

### Unintended consequences

One potential unintended consequence related to the possibility for the genogram to create distress. One woman expressed concerns of how the genogram could have negative impacts, particularly amongst women who were potentially more vulnerable, i.e. teenagers, by highlighting a lack of available support:

I think if you were a, I don’t know 17 year old girl with very little support it could be … but it could be good because it could give them avenues, people who they could speak to, so it could put them in touch with these community centres and stuff like that. But it could also show that they are very much on their own, so it could have the opposite effect. (P2, Site A)

Some Site A IFHs raised concerns that the genogram could serve as a *‘concrete’* reminder of women’s limited support networks and how *‘putting that down on a piece of paper is actually quite soul destroying’.* One IFH also described a situation when completing the genogram was not appropriate due to the woman’s difficult life circumstances:

We didn’t do it with the first lady, I explained it and then she burst into tears, and I was like, “I’m so sorry,” and she said, “My dad just died and my mum lives in [place] and she has disowned me, and my aunt keeps going on about bottle feeding, can we do it another time?” I was like, “Yeah that’s fine.” But she never did it. (IFH 8, Site B, Focus Group)

Some of the conversations and discussions stimulated by the genogram were not always positive. For instance, for one woman an infant feeding discussion with her mother had led to her feeling *‘disgruntled’* when it transpired that her belief of being breastfed was incorrect.

A further unexpected consequence, but from a positive perspective, concerned how genogram completion served to form a connection between the IFH and woman. One IFH referred to how she would use the information in the genogram to show value and to develop a trust-based mother-helper relationship:

I didn’t keep the actual diagrams, but I did take a picture on the phone so that I could remember the names and things. I just wanted them to feel valued really and that they could trust me and speak to me if they needed to really. (IFH 13, Site B, Interview)

## Discussion

In this paper we report on women’s and IFHs’ views and experiences of an infant feeding genogram delivered within an assets-based peer support feasibility trial. Content analysis of completed genograms and the qualitative accounts highlighted variation in the IFHs application of the tool across the two sites. These insights illustrate how the genogram was either used as intended to reinforce and/or extend women’s social connections and support, or was utilised as a data collection tool, with limited perceived utility to mothers. The genogram also had the potential to cause unintended consequences such as magnifying a lack of immediate support or encouraging access to support that was deemed unhelpful or helped to forge positive mother-helper relationships.

A strength of this study is that it is the first time a genogram has been used as an intervention tool with the aim of facilitating an assets-based approach to infant feeding. Content analysis of the infant feeding genogram, together with qualitative insights offered triangulation to explore and critique women’s and IFHs’ experiences. Purposive sampling also meant we captured the views of women with different backgrounds and levels of engagement with the ABA intervention. We could have undertaken a triangulated analysis where we just focussed on women’s and helpers’ views of completed genograms (as available). However, this would have only provided partial insights, as, e.g. some of the more negative views of genogram completion were from those who did not provide any completed genograms. Our inclusive approach meant we were better able to understand how and why the genograms were being used in practice. As the focus groups/interviews explored the ABA intervention, with the genogram being just one component, this may have restricted the insights generated. Furthermore, the variations in the length of the postnatal period at time of interview may also have influenced women’s responses, e.g. in the utility of the genogram on help-seeking behaviours at different time points.

The ABA intervention was underpinned by two core-BCTs which were delivered through the genogram activities namely ‘restructuring the social environment’ and ‘social support (unspecified)’ [24, 25]. Findings indicate that the performance of the genogram enhanced awareness of available support for some women, impacting on their motivation and confidence to take advantage of these assets and to seek support for their infant feeding behaviours. These findings support those by Darwent et al. [20] and are in line with the COM-B model [25] suggesting that genogram use elicits perceptions of social opportunities, motivation and capability, thereby increasing the likelihood of behavioural performance. However, results indicate that the perceived impact of the genogram may be closely related to the IFH’s application of the tool. The variation on genogram application seemed to be related to IFH’s views and perceptions of value and usefulness; with differences noted between Site A and Site B, despite receiving the same training, albeit on different occasions. Overall, Site B participants held more positive views on the genogram, which in turn translated into positive engagement with the tool by women. Broadly, at Site B the IFHs appeared to be aware of the tool’s purpose, and to demonstrate tool fidelity. Site B genograms were more personal, individualised and provided richer detail (Types 3 and 4). In contrast, at Site A the IFHs were less likely to use the tool as intended. This was reflected in genograms that contained impersonal and basic information, and in accounts that suggested the genogram was used to collect data, rather than the basis of a meaningful infant feeding discussion (Types 1 and 2). While it is important to reflect that not all IFHs/women on Site A were negative and not all Site B IFHs/women were positive, the broad distinction between the use of the genograms between the sites suggests that genogram completion is a tool to facilitate a meaningful helper-woman relationship rather than a proxy that can stand in the stead of those relationships.

The variations in genogram use support the premise that assets can be leveraged and utilised, but how and if they are used depends on the individual [28]. The differences in IFH application of the tool may relate to their different backgrounds and duration since they commenced as a peer supporter, which was generally longer in Site A. Site A peers were employed breastfeeding peer supporters with work related targets, i.e. increases in breastfeeding rates, prior to becoming ABA helpers. The fact that a number of Site A IFHs struggled to provide individualised and balanced infant feeding information may reflect the findings of Aiken & Thomson [29]. These authors report on how the professionalisation of peer support through enforced accountabilities can be to the detriment of providing in-depth, woman-led support. Assets-based methods operate to situate individuals as co-producers of health [15] – our findings suggest that some IFHs, particularly those at Site A struggled with this egalitarian approach. As Site B IFHs were breastfeeding volunteers, a role generally underpinned by altruistic intentions to make a difference to women’s experiences [29], this may explain why adoption of the asset-based approach was more readily embraced. While the genogram has been highlighted as requiring minimal training [30], the input provided in this study was very limited. To make full use of the tool, IFHs may need training not only in practical techniques but also in the facilitation and listening skills that change it from a data recording tool to one with therapeutic/asset generating value. A work-related incentive for peers working within a paid service, such as management recognition, may also provide further motivation [31]. While the genogram had the potential to cause negative impacts by highlighting a lack of available support, focused training would help to re-envision this situation as an opportunity to empower women via strengthening and extending their supportive networks. Furthermore, as there were issues across both sites about continued use of the genogram during postnatal contacts, further training such as role plays to highlight its ongoing value, as well as a digital version of the genogram (e.g. shared via WhatsApp) for ease of access may prove beneficial.

Some IFHs used the women’s personal information collected during genogram construction - such as the names and backgrounds of their supporters - to demonstrate value and to direct women to needs-led care. These insights thereby highlight how the tool could promote continuity and individualised care, which reflects the expectations of the *Better Births* agenda [32]. The UNICEF-UK Baby Friendly initiative has recently changed its approach to a focus on mother and infant relationships, and where support is contextualised by a mother’s lived realities and with an emphasis on the importance of ‘meaningful conversations’ with parents about their feeding decisions [33]. The genogram with its woman-centred, context related approach aligns well with this ethos, and could be a welcome addition for midwives, and peer/lay supporters to help prepare women for the realities of infant feeding [20].

## Conclusion

This paper demonstrates how a genogram in a novel health care research context can stimulate a meaningful conversation with women about their infant feeding history and sources of available support. It could help women reframe help-seeking as a strength, and identify new and unexpected sources of support, strengthening their social connectedness. The use and impact of the genogram is associated with the attitude, skills and confidence of the IFH, with more sophisticated and useful diagrams being produced by IFHs who used a woman-centred, embodied approach. How the genogram is valued and communicated is critical. Additional training, supervision and mentoring may be required both in tool use but additionally in the generic competencies such as listening and facilitation. Notwithstanding this requirement, this study highlights that using an infant feeding genogram has the potential to change the focus of women-professional interactions to a more woman, family and community-centred approach that focusses on building intrinsic and extrinsic assets for infant feeding.

## Supplementary information

**Additional file 1: Supplementary file 1.** Interview schedule – Women.

**Additional file 2: Supplementary file 2.** Focus group/interview schedule – Infant Feeding Helpers.

## Data Availability

All key data concerning this work is included in the manuscript. Further anonymised quotes are available subject to reasonable request.

## Abbreviations

ABA: Assets-based infant feeding help Before and After birth feasibility trial
BCT: Behaviour Change Techniques
IFH: Infant Feeding Helper
