# Supplementary material for: Exploring the use and experience of an infant feeding genogram to facilitate an assets-based approach to support infant feeding
Source: BMC Pregnancy Childbirth. 2020 Sep 29;20:569. doi: 10.1186/s12884-020-03245-8 (PMC7523065; doi:10.1186/s12884-020-03245-8)
Supplement: Supplementary file 1 — Additional file 1: Supplementary file 1. Interview schedule – Women. [file 12884_2020_3245_MOESM1_ESM.docx]

**Supplementary File 1: Interview schedule - Women**

**Questions for All participants / *Questions for ABA participants only***

**the mother’s Feeding story**

1. **Thank you so much for taking the time to talk to me. Can I start by asking how old your baby is now … baby’s name, how are you both?**
    [make a note of baby’s name for use in rest of interview]
2. **Can you tell me about your experience of feeding your baby …?**

[Encourage mother to tell her story]

**Were there any challenges or difficult times in terms of feeding your baby?**

(If so) What did you do?

(If so) Who helped you?

How are things now?

**EXPERIENCE OF ANTENATAL FEEDING HELP**

**Thinking back to before your baby was born …** how were you thinking about feeding your baby?

How different is your experience to what you had expected?

Is there anything they would say to friends who are pregnant for the first time to help them prepare?

1. ***Thinking back to the first time you met your ABA helper, before your***

***baby was born… can you tell us what happened – what did you talk about? What was helpful/unhelpful?***

1. ***Did you any receive text messages or phone calls from the ABA helper***

***before your baby was born?*** *(If so) what did you think about the messages and calls that you received?*

1. ***Did you and the ABA helper talk about how friends/family members had fed their babies?*** *(If so) Did you find that helpful? How / Why?*
2. ***Did your ABA helper provide you with any information about local groups or where to get support?*** *If so, can you tell me about any support have you accessed?*
3. ***To what extent did help from ABA influence how you were thinking of feeding your baby?***

**EXPERIENCE OF POSTNATAL FEEDING HELP**

1. **Can you tell me about your experience of infant feeding help in the hospital?** Who provided it, useful/not useful?
2. ***Can you talk me through what happened after the birth with the ABA feeding helper?***

*Who contacted who, what happened next?*

1. ***Can you tell me about what ABA help you received?***

*How did you organise how often she would contact you – how did she support you –what it was like – was it enough?*

1. **Can you tell me about other types of help you have received for infant feeding – so any help you have received from health professionals, friends, family, other support?**

What was helpful/unhelpful?

1. **Can you tell me about any times when you particularly needed help with feeding your baby – what happened?**
2. **Thinking about the help that you got from family and friends, were there any costs involved?**
   E.g. Did they take unpaid time off work? Pay or travel to attend groups? Buy equipment?
3. ***Did any of the midwives or health visitors that you spoke to mention the ABA service?****(If so) What did they say about it?*

***RELATIONSHIP WITH THE ABA HELPER***

1. ***How would you describe your relationship with the ABA helper?***
2. ***Did your relationship with the ABA helper change over time? If so how***
3. ***Can you tell me about any ways in which the ABA helper has influenced you or your experience of feeding your baby?***

*Explore answer*

1. ***Thinking about being part of the ABA study, have you talked to friends or family about it?***

*Can you tell me about some of the conversations you have had? What have been their thoughts about it?*

**CONTAMINATION / COMMUNITY LEVEL EFFECT**

1. **Have you met any (other) mothers who were taking part in the ABA study? (If so) did you meet any mothers who saw an ABA infant feeding helper? Did they talk about the help they got from the ABA infant feeding team with you?**(If so) what did they say?

Did they pass on any ideas or tips or information about ways to get help?

(If so) Did the information help you?

**FINAL THOUGHTS**

1. **Thinking about immediate family, friends, health professionals and anyone else who has been around … who do you feel has been most helpful to you with feeding your baby?**Who … why/ how?
2. ***Is there anything you would change about ABA?***
3. **Do you have any other issues or views you wish to share about your experiences?**

**Thank you for your time**
