# Supplementary material for: Exploring the use and experience of an infant feeding genogram to facilitate an assets-based approach to support infant feeding
Source: BMC Pregnancy Childbirth. 2020 Sep 29;20:569. doi: 10.1186/s12884-020-03245-8 (PMC7523065; doi:10.1186/s12884-020-03245-8)
Supplement: Supplementary file 2 — Additional file 2: Supplementary file 2. Focus group/interview schedule – Infant Feeding Helpers. [file 12884_2020_3245_MOESM2_ESM.docx]

**Supplementary File 2: Focus group/interview schedule – Infant Feeding Helpers**

**PEER EXPERIENCE AND UNDERSTANDING**

1. **What do you understand are the goals of the ABA intervention?**

How do you feel about these goals?

1. **How did being an ABA feeding helper differ from the help you used to/usually provide?**

**ANTENATAL DELIVERY**

1. **Thinking about the first time you met the mothers face-to-face, before their babies were born, how did that go?**

Prompts: How was it arranging a time and place to meet? Anyone else there?

Any difficulties/challenges?

1. **Thinking more about those first face-to-face meetings, how did you find discussing the mothers’ feeding views ?**

How did ABA fit with mothers’ feeding views?

1. **How did you find using the family and friends tree (Genogram)?**

Prompts: How useful was it, any difficulties, what influence do you think it had (e.g. women seeking out support), any suggestions for using this in future?

1. **How did you pass on information about local groups and other sources of help?**

Prompts: How was that received? How useful were they?

1. **How did you find texting and making calls to the mothers before their babies were born?**

Prompts: What worked well - didn’t work well?

1. **Did you accompany any of the mothers you met to a local group before her baby was born?**

Prompts: If no, why not? If yes, how was it?

**POSTNATAL DELIVERY**

1. **Now, thinking about after the babies were born, how did you find making contact with the women?**

Prompts: How did you organise the frequency of contacts with mothers? Were you able to organise face-to-face contacts/accompany women to groups (if not why – if yes, how was it); What worked well – didn’t work well?

1. **How did you find texting and making calls to the mothers after their babies were born?**

How did the mothers respond?

**IMPACT**

1. **When did women most need help?**

To what extent do you feel you were able to provide help when women most needed it?

**12. How did the ABA infant feeding support influence women asking for help from others?**

1. **How do you think ABA influenced women?**

**INTEGRATION**

1. **What was your experience of working alongside health professionals as part of the ABA intervention?** (explore answer)

1. **How did you feel about ABA being available to some mothers but not others?**

Prompts: How did that work out in practice? Were you asked to support women not in the ABA group?

**EXPERIENCE OF TRAINING AND SUPERVISION**

1. **What are your thoughts about the ABA training?**

Prompts: What was good – not so good?

What did you learn that was new? What would you do differently?

1. **Will the training change the way you help mothers in the future once the ABA study has ended? If so, how?**

**FINAL THOUGHTS**

1. **Overall, what has been your experience of being an ABA feeding helper?**

Prompts: Anything you would you have liked to do differently? Aspects you disagreed with?; Did you make any changes to how the ABA support was meant to be provided (explore answer)

1. **Did the ABA intervention have any additional costs for you?**

For example, longer unpaid hours, cost of childcare, cost of telephone calls?

1. **If in future ABA was to become part of usual care – what might be the issues?**
2. **In your opinion, do you think it would be helpful to roll out the ABA intervention to all mothers?** Why/ Why not?
3. **Do you have any other issues or views you wish to share about the ABA intervention?**

**Thank you for your time**
